# Improving timing resolution of BGO for TOF-PET: a comparative analysis with and without deep learning

**DOI:** 10.1186/s40658-024-00711-6

**Published:** 2025-01-17

**Authors:** Francis Loignon-Houle, Nicolaus Kratochwil, Maxime Toussaint, Carsten Lowis, Gerard Ariño-Estrada, Antonio J. Gonzalez, Etiennette Auffray, Roger Lecomte

**Affiliations:** 1 Instituto de Instrumentación para Imagen Molecular, Centro Mixto CSIC-Universitat Politècnica de València, Camino de Vera, Valencia, 46002 Spain; 2 Department of Biomedical Engineering, University of California Davis, One Shields Ave., Davis, California 95616 USA; 3 CERN, Department EP-CMX, Esplanade des Particules 1, Meyrin, 1217 Switzerland; 4 Sherbrooke Molecular Imaging Center and Department of Nuclear Medicine and Radiobiology, Université de Sherbrooke, 12th Avenue N, Sherbrooke, J1H 5N4 Québec Canada; 5 RWTH Aachen University, 55 Templergraben, Aachen, 52062 Germany; 6 Institut de Fìsica d’Altes Energies, Barcelona Institute of Science and Technology, Edifici Cn, Campus UAB, Bellaterra, Barcelona, 08193 Spain; 7 Imaging Research and Technology (IR&T) Inc., 2201 Tanguay St., Magog, Québec J1X 7K3 Canada

**Keywords:** Time-of-flight PET, Fast timing, BGO, Time resolution, Cherenkov, Deep learning

## Abstract

**Background:**

The renewed interest in BGO scintillators for TOF-PET is driven by the improved Cherenkov photon detection with new blue-sensitive SiPMs. However, the slower scintillation light from BGO causes significant time walk with leading edge discrimination (LED), which degrades the coincidence time resolution (CTR). To address this, a time walk correction (TWC) can be done by using the rise time measured with a second threshold. Deep learning, particularly convolutional neural networks (CNNs), can also enhance CTR by training with digitized waveforms. It remains to be explored how timing estimation methods utilizing one (LED), two (TWC), or multiple (CNN) waveform data points compare in CTR performance of BGO scintillators.

**Results:**

In this work, we compare classical experimental timing estimation methods (LED, TWC) with a CNN-based method using the signals from BGO crystals read out by NUV-HD-MT SiPMs and high-frequency electronics. For ${2 \times 2 \times 3}\,\hbox {mm}^{3}$ crystals, implementing TWC results in a CTR of 129 ± 2 ps FWHM, while employing the CNN yields 115 ± 2 ps FWHM, marking improvements of 18 % and 26 %, respectively, relative to the standard LED estimator. For ${2 \times 2 \times 20}\,\hbox {mm}^{3}$ crystals, both methods yield similar CTR (around 240 ps FWHM), offering a $\sim$15 % gain over LED. The CNN, however, exhibits better tail suppression in the coincidence time distribution.

**Conclusions:**

The higher complexity of waveform digitization needed for CNNs could potentially be mitigated by adopting a simpler two-threshold approach, which appears to currently capture most of the essential information for improving CTR in longer BGO crystals. Other innovative deep learning models and training strategies may nonetheless contribute further in a near future to harnessing increasingly discernible timing features in TOF-PET detector signals.

## Background

Rapid advances in positron emission tomography (PET) are currently made possible by deep learning methods, whether in detectors, data acquisition or image reconstruction [1]. Combined with ultrafast time-of-flight (TOF), another topic of recent high interest in PET [2–4], deep learning approaches promise to harvest more information from detected signals to increase image signal-to-noise ratio (SNR) and improve image quality, thereby enhancing diagnostic accuracy. Improvements of the whole detection chain—scintillators, photodetectors and electronics—can boost the coincidence time resolution (CTR) of detectors that is needed for accurate TOF measurement [5, 6]. These detector improvements can also bring out higher quality and sharper features underlying the interaction process, which could then be exploited to further improve the CTR.

Many state-of-the-art CTR values upon 511 keV irradiation were established in [7] for multiple scintillation detectors coupled to NUV-HD SiPMs by Fondazione Bruno Kessler (FBK) [8, 9] and new high-frequency (HF) electronics [10, 11]. Improvements of these SiPMs by FBK have been achieved by using metal-trench (MT) technology which reduces the SiPM crosstalk [12] and therefore has pushed the CTR values further [13]. These CTRs were obtained using a leading edge discrimination (LED) estimator recording the signal crossing time at a threshold on the rising edge of the waveforms. Improvement of the CTR of bismuth germanate (BGO) crystals was demonstrated by using event classification with a second higher threshold to perform time walk correction (TWC) [14–16]. Recently, improved CTR was obtained for BGO by using newly developed SiPMs from FBK with improved single photon time resolution (SPTR) [17]. Other timing estimation techniques using more than one or two threshold(s) were also explored in the past years, with the goal of retrieving more information from signals of various PET detectors. These techniques include maximum likelihood methods [18, 19], investigation of depth-of-interaction influence on CTR using two thresholds [20], or even using a Gauss-Markov estimator on series of timestamps assigned by fully digital photodetectors [21].

With the emergence of deep learning recently thriving in many fields—PET instrumentation being no exception—new deep learning–based TOF estimation techniques were proposed. Compared to a classical LED estimation, the CTR of lutetium fine silicate crystals and photomultiplier tubes (PMTs) was improved by $\sim$20 % with a convolutional neural network (CNN) trained on coincident waveforms measured for different positions of a radioactive source [22]. Recently, a CNN was also used for TOF estimation in a direct, reconstruction-free positron imaging with CTR close to 30 ps full width at half maximum (FWHM) [23]. This demonstration was made for thin Cherenkov radiators coupled to microchannel plates PMTs, requiring long acquisition times because of their low sensitivity to 511 keV photons. Another TOF estimation method was proposed by training a CNN using digitized waveforms measured at a single source position and combining the information of the CNN together with the LED, showing CTR improvement of $\sim$10 % for LYSO crystals coupled to multi-pixel photon counters [24].

In light of all these recent works, it can be seen that more precise TOF estimation for enhanced CTR can be achieved by exploiting information on the rising edge of waveforms. This information content is especially present in the case of BGO with Cherenkov light, where a few prompt photons might be detected on top of the slow scintillation response, yielding to event-to-event differences [14]. A lower-complexity approach uses two thresholds to measure the signal rise time to perform TWC, whereas more complex approaches require finer digitization of the waveforms to train CNNs.

In this study, we aim to compare a TWC-based approach and a CNN-based approach. A novelty of the present work is to explore CNN-based TOF estimation for signals obtained with an HF readout [10, 11] and new NUV-HD-MT SiPMs [12]. We focus the analysis on BGO with Cherenkov photons, since detector concepts based on Cherenkov light [25–28] are seen as a potential replacement for the L(Y)SO crystals in TOF-PET scanners because of their lower cost, higher stopping power and efficient TWC [15, 17, 29]. By comparing the outcomes of using TWC- or CNN-based approaches, our study intends to show the relative merits and limitations of methods with and without deep learning for improving time resolution.

## Methods

### Detectors and experimental setup

A ^22^Na radioactive source (511 keV) was placed between two detectors made of teflon-wrapped BGO crystals coupled with Meltmount glue ($n=1.58$) to 4x4 mm NUV-HD-MT SiPMs from FBK and biased at 48 V ($V_{\text {breakdown}} = {32\,\textrm{V}}$). Two measurement runs were performed, each time with two detectors of the same type in coincidence positioned $\sim$8 cm apart: 1) BGO of ${2 \times 2 \times 3}\,\hbox {mm}^{3}$ and 2) BGO of ${2 \times 2 \times 20}\,\hbox {mm}^{3}$. A high-frequency electronic readout [10, 11] was used to obtain the time signal together with an analog operational amplifier for the energy signal. The signals were digitized using a LeCroy DDA735Zi oscilloscope (bandwidth of 3.5 GHz) at a sampling rate of 20 Gs $\hbox {s}^{-1}$ and interpolated by the oscilloscope with a $\sin (x)/x$ function, giving a 5 ps time step. Also with this fine digitization, a 150 mV voltage scale was used to focus on the initial part of the rising edge at which the HF readout enhances the timing resolution. The waveforms were recorded to be analyzed offline. The energy signals were integrated to create energy spectra from which only photoelectric events were kept—within 440 to 665 keV as in [14]—after fitting with a Gaussian and a linear functions on the photopeak and Compton edge. The crossing times were recorded at two thresholds (10 mV and 100 mV) in each detector. The first threshold value was found to provide good results in BGO in previous works utilizing a similar experimental setup [14, 15], whereas the second threshold was higher compared to previous works of only 50 mV to account for the increased SiPM gain at the high overvoltage with the new NUV-HD-MT technology. The amplitude of a single cell is about 90 mV, hence the second threshold is above the one-photon level, and the choice of the two thresholds is not the most crucial aspect as long as fluctuations have sufficient time to propagate [14, 30]. The difference of the crossing times between the two detectors at the lower threshold, which we used for determination of time delay between the two detectors and call coincidence time delay denoted by $t_{\text {led}}$, was evaluated for each coincidence event. The rise time, denoted by $t_{\text {rise}}$, in each detector was evaluated by the time difference between the crossing times at the high and low thresholds. Events with a coincidence time delay beyond specified coincidence time windows (1 ns and 2 ns for BGO ${2 \times 2 \times 3}\,\hbox {mm}^{3}$ and ${2 \times 2 \times 20}\,\hbox {mm}^{3}$, respectively) and/or exhibiting an unstable baseline—where the voltage exceeds $2\sigma$ outside the mean baseline within a nanosecond region ending 0.5 ns before the rising edge—have been excluded from the dataset. This gave 36975 and 21766 valid events for BGO ${2 \times 2 \times 3}\,\hbox {mm}^{3}$ and BGO ${2 \times 2 \times 20}\,\hbox {mm}^{3}$, respectively. More details of the experimental setup can be found in [15].

### Waveform processing and CNN architecture

**Fig. 1:**
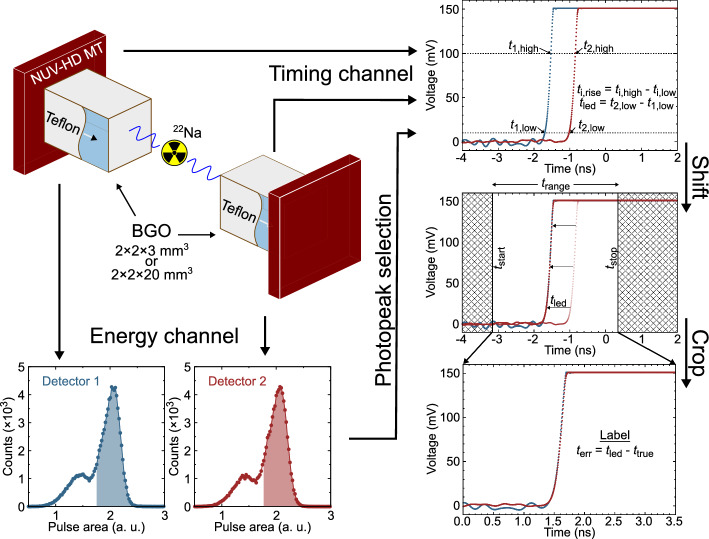
Schematic of the experimental waveform acquisition and pre-processing, showing the photopeak selection, the measurement of crossing times at 10 mV and 100 mV to evaluate both the coincidence time delay ($t_{\text {led}}$) and rise time ($t_{\text {rise}}$ in both detectors), and the shifting and cropping of the waveforms for the CNN as proposed in [24]. The waveforms are saturated because a focus was made on the early part of the rising edges on the oscilloscope

Preprocessing of the waveforms for the CNN was first done to keep a time window around their rising edges. The procedure is fully detailed in [24] and briefly exposed here, supported by Fig. 1.

For each coincidence waveform pair, one of the waveform was shifted by $t_{\text {led}}$ to align the waveforms. There is therefore an estimation error ($t_{\text {err}}$) of the LED compared to the true TOF ($t_{\text {true}}$) defined by the physical source position:

1 $$\begin{aligned} t_{\text {err}}= t_{\text {led}}- t_{\text {true}}. \end{aligned}$$

The labels to train the CNN were the $t_{\text {err}}$ values. The labels therefore formed a continuous distribution centered at zero. This eliminates the bias at the edges of a training space that would occur with a discrete label distribution made, for instance, with TOF values of multiple discrete source positions. The incorporation of the LED into the CNN training to obtain a continuous label distribution that eliminates the bias was first suggested by Onishi *et al.* [24]. A time range of 3.5 ns for each waveform was kept. The starting time was fixed at 1.5 ns before the earliest waveform crossing the LED threshold, in order to capture some baseline noise. After the waveforms were shifted and cropped, they were normalized with a min-max normalization and served as inputs for the CNN where each coincidence waveform pair had a size of $2\times 700$. This fine stepping was used to evaluate the achievable timing limits of BGO. The waveform dataset was split 65 %/5 %/30 % in training, validation and testing sets, respectively.

A five-layer network architecture was implemented: three convolutional layers were followed by a fully connected layer with 256 output neurons and a final fully connected layer with single neuron output for TOF regression. A schematic of the CNN from which the current architecture was inspired is shown in [24]. The first convolutional layer used a 2x5 kernel (32 filters), then the two next convolutional layers both used a 1x3 kernel (64 filters). Except for the last fully connected layer, each layer in the CNN was followed by a ReLU activation function. Max pooling (size of 1x3) was done after each ReLU activation of the three convolutional layers. For BGO ${2 \times 2 \times 3}\,\hbox {mm}^{3}$ (${2 \times 2 \times 20}\,\hbox {mm}^{3}$), the learning rate was fixed at e-4 (e-5) then twice decreased by a factor ten at 30 % and 60 % of the total of 100 epochs. The CNN was trained using the Adam optimizer with a mean square error loss function and minibatches of 32 waveforms. Details of this optimization to perform the TOF estimation is given in Sec. 2.3.

### TOF estimators

Three different methods to perform the timing estimation were compared: 1) leading-edge discrimination, 2) leading-edge discrimination with time-walk correction and 3) CNN joined with leading-edge discrimination. These three methods will be referred to as LED, TWC and CNN, respectively.

For the LED method, the $t_{\text {led}}$ values were directly used as the timing estimator. For the TWC method, a scatter plot of $t_{\text {led}}$ against $t_{\text {rise}}$ for each of the two detector channels was drawn and a linear function was fitted in both plots. Then, the coefficients of the fit can be used to correct each $t_{\text {led}}$ for the left and right (*l*, *r*) detector channels

2 $$\begin{aligned} \hat{t}_{\text {twc}, i} = t_{\text {led}, i} - \frac{1}{2} \sum _{j \in \{l, r\}} \left( p_{0,j} + p_{1,j} \cdot t_{\text {rise}, j} \right) \end{aligned}$$

where $t_{\text {led}, i}$ is the LED coincidence time delay of event *i*, $p_{0}$ and $p_{1}$ are the intercept and slope of the fit and $t_{\text {rise}}$ is the rise time. This correction method is fully detailed in [15].

For the CNN method, the timing estimation was done in accordance to the method in [24], which also uses the LED information to have unbiased estimation. The CNN output, denoted $t^*$, was used to perform the TOF estimation as $t_{\text {est}}= t_{\text {led}}- t^*$. This therefore corresponds to a correction of the time difference error since $t_{\text {led}}$ can be replaced by $t_{\text {true}}+ t_{\text {err}}$ from Eq. (1), giving $t_{\text {est}}= t_{\text {true}}+ t_{\text {err}}- t^*$. This last equation indicates that an ideal case with perfect correction would give the true TOF. We also remind that a powerful aspect of this implementation approach is that the CNN only needs training data obtained with one source position (i.e., at a single $t_{\text {true}}$ value) and the trained CNN can provide a TOF estimation for other source positions having different $t_{\text {true}}$ [24].

### Timing performance evaluation

Since the trained CNN must be evaluated with waveforms that were not used during the training, only the test dataset was used to assess its performance. So, even though no training was done with the LED and TWC methods, the same test dataset was used for CTR evaluation to have a direct performance comparison with the CNN method. Because of the finite number of waveforms, the performance for all three methods can however depend on the content of this test dataset (and consequently also the training set for the CNN method). To better gauge the performance variability of the methods, we used bootstrapping—with 50 resampling—to produce different random sampling of the full waveform dataset to create multiple splitting into the training, validation and test datasets. This enables the estimation of the CTR characteristics (e.g., mean and variance) for all three methods.

For each of the resampling, the timing values provided by each method were histogrammed and fitted with two Gaussian functions [14, 15]

3 $$\begin{aligned} k_{(x)} = \frac{N}{\sqrt{2\pi }} \cdot \left[ \frac{r_C}{\sigma _C} \cdot \exp \left( -\frac{(x-\mu )^2}{2\sigma _C^2} \right) + \frac{1-r_C}{\sigma _S} \cdot \exp \left( -\frac{(x-\mu )^2}{2\sigma _S^2} \right) \right] \end{aligned}$$

where the first and second parts in brackets represent the temporal delay primarily attributed to Cherenkov photons (standard deviation of $\sigma _C$ and abundance $r_C$) and scintillation photons (standard deviation of $\sigma _S$ and abundance $1-r_C$), respectively, with the number of events *N*. The FWHM CTR (denoted $\hbox {CTR}_{\text {FWHM}}$) and FWTM CTR (denoted $\hbox {CTR}_{\text {FWTM}}$) were then evaluated from the fitted function. An important aspect to note is that a double Gaussian fit may have greater instability than a single Gaussian fit, resulting in more potential solutions and less precision in determining exact component parameters. The reason for this lies in the correlation between individual fit values; a lower abundance of the narrow Gaussian may result in a smaller width, and vice versa.

Furthermore, a formalism to evaluate an equivalent conventional TOF resolution in the case of non-Gaussian TOF kernels was recently proposed [31], therefore useful for BGO with its double Gaussian kernel. The formalism relies on the particular PET imaging task of detecting a hot spot within the middle of a uniform cylinder, followed by the subsequent reconstruction of the central voxel. This was proposed for two situations, whether it is possible or not to label each event with its own TOF kernel, which affects how the SNR is computed. In the case it is not possible (unlabeled case), the equivalent resolution is given by

4 $$\begin{aligned} \sigma _{\text {eq, unlabeled}} = \frac{1}{2\sqrt{\pi }\int _{-\infty }^{\infty }k^2(x)dx} \end{aligned}$$

where *k*(*x*) is the TOF kernel, which in our case is the fitted double Gaussian function used for BGO. Labeling the events with their appropriate TOF kernel can help improve the SNR, and the equivalent resolution is given by

5 $$\begin{aligned} \sigma _{\text {eq, labeled}} = \left( \frac{r_C}{\sigma _C} + \frac{1-r_C}{\sigma _S}\right) ^{-1}. \end{aligned}$$

Approximations done in [31] are considered as valid and for reasons of simplicity, the PET scanner diameter and the source activity are set to unity. We primarily used the unlabeled equivalent TOF resolution to compare the performance of the three methods. We also evaluated the gain in time resolution in the case where labeling of the events would be available. In both cases (Eq. (4) and (5)), the equivalent TOF resolution was multiplied by a factor 2.355 for conversion to FWHM, and the resulting value is referred to as an equivalent CTR (denoted $\hbox {CTR}_{\text {eq}}$).

## Results

**Fig. 2:**
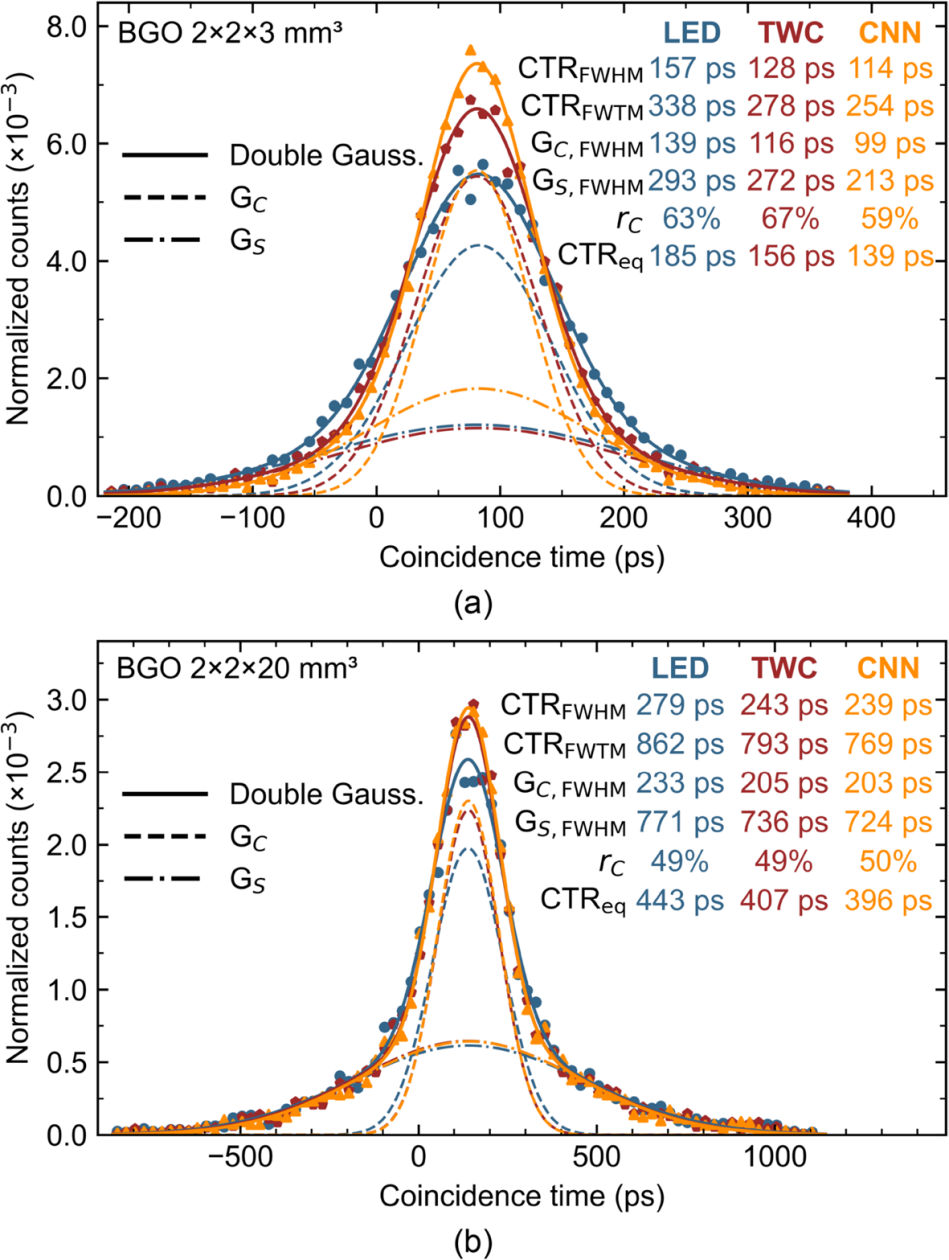
Coincidence time distributions for (a) BGO ${2 \times 2 \times 3}\,\hbox {mm}^{3}$ and (b) BGO ${2 \times 2 \times 20}\,\hbox {mm}^{3}$. The dashed and dash-dotted lines represent the components ($\hbox {G}_C$ and $\hbox {G}_S$ in the legend) of the double Gaussian fitting function (solid line) in Eq. (3). The $\hbox {CTR}_{\text {eq}}$ values correspond to unlabeled TOF events (Eq. (4))

Examples of coincidence time histograms for the LED, TWC and CNN methods—using the sampled dataset giving the closest $\hbox {CTR}_{\text {FWHM}}$ values to the averages of all samples obtained with the bootstrap (see Sec. 2.4)—are shown in Fig. 2 for the two BGO geometries. The higher gain, e.g., the sharper time histogram, of CNN compared to TWC can be appreciated for BGO ${2 \times 2 \times 3}\,\hbox {mm}^{3}$, whereas similar histograms are seen for BGO ${2 \times 2 \times 20}\,\hbox {mm}^{3}$.

**Fig. 3:**
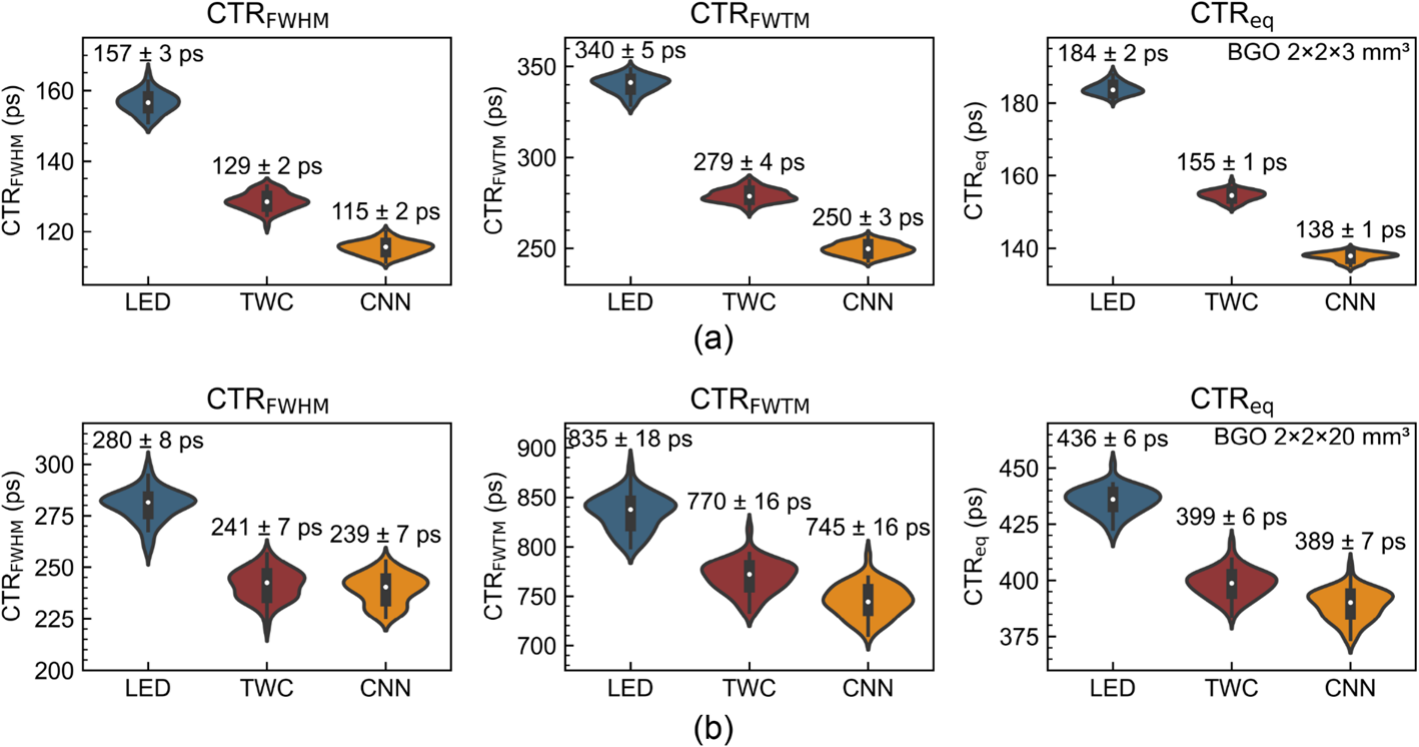
Violin plots of the $\hbox {CTR}_{\text {FWHM}}$, $\hbox {CTR}_{\text {FWTM}}$ and $\hbox {CTR}_{\text {eq}}$ (unlabeled version, Eq. (4)) distributions for the three methods for (a) BGO ${2 \times 2 \times 3}\,\hbox {mm}^{3}$ and (b) BGO ${2 \times 2 \times 20}\,\hbox {mm}^{3}$. The average and standard deviation is shown above each distribution

The full CTR performance of the timing methods is shown in Fig. 3 for the two BGO dimensions. For BGO ${2 \times 2 \times 3}\,\hbox {mm}^{3}$, the measured $\hbox {CTR}_{\text {FWHM}}$ for LED is 157 ± 3 ps, which improves to 129 ± 2 ps for TWC (18 % gain) and 115 ± 2 ps for CNN (26 % gain). The two other metrics ($\hbox {CTR}_{\text {FWTM}}$ and $\hbox {CTR}_{\text {eq}}$) have improvements in the same range. For BGO ${2 \times 2 \times 20}\,\hbox {mm}^{3}$, the measured $\hbox {CTR}_{\text {FWHM}}$ for LED is 280 ± 8 ps, which improves to 241 ± 7 ps for TWC (14 % gain) and 239 ± 7 ps for CNN (15 % gain). The CNN therefore yields similar gain as TWC, although reduced tails with the CNN enables a slightly better improvement in $\hbox {CTR}_{\text {FWTM}}$ and $\hbox {CTR}_{\text {eq}}$. Some asymmetry is observed in the distributions of Fig. 3 which could be due to the bootstrapping method that deals with a limited re-sampled numbers of waveforms that can increase the variability of the CTR, together with some inherent instabilities in fitting double-Gaussian distributions. A summary of all the results of CTR performance and improvement for the TWC and CNN methods against the LED method is given in Table 1.

**Table 1:** Measured $\hbox {CTR}_{\text {FWHM}}$, $\hbox {CTR}_{\text {FWTM}}$ and $\hbox {CTR}_{\text {eq}}$ using LED, TWC and CNN

Scintillator	Method	$\hbox {CTR}_{\text {FWHM}}$		$\hbox {CTR}_{\text {FWTM}}$		$\hbox {CTR}_{\text {eq}}$	
		(ps)	Gain (%)	(ps)	Gain (%)	(ps)	Gain (%)
BGO ${2 \times 2 \times 3}\,\hbox {mm}^{3}$	LED	157 ± 3	-	340 ± 5	-	184 ± 2	-
	TWC	129 ± 2	18 ± 2	279 ± 4	18 ± 2	155 ± 1	16 ± 1
	CNN	115 ± 2	26 ± 2	250 ± 3	27 ± 1	138 ± 1	25 ± 1
BGO ${2 \times 2 \times 20}\,\hbox {mm}^{3}$	LED	280 ± 8	-	835 ± 18	-	436 ± 6	-
	TWC	241 ± 7	14 ± 4	770 ± 16	8 ± 3	399 ± 6	8 ± 2
	CNN	239 ± 7	15 ± 3	745 ± 16	11 ± 3	389 ± 7	11 ± 2

The gain values are all in relative to the LED method. A gain is defined in terms of improvement, thus a lower CTR value compared to LED yields a higher gain. Errors represent a 1$\sigma$ interval using the distribution of CTR values obtained by the bootstrapping process, or from error propagation on the gain calculation

^1^Computed with the unlabeled version (Eq. (4))

**Fig. 4:**
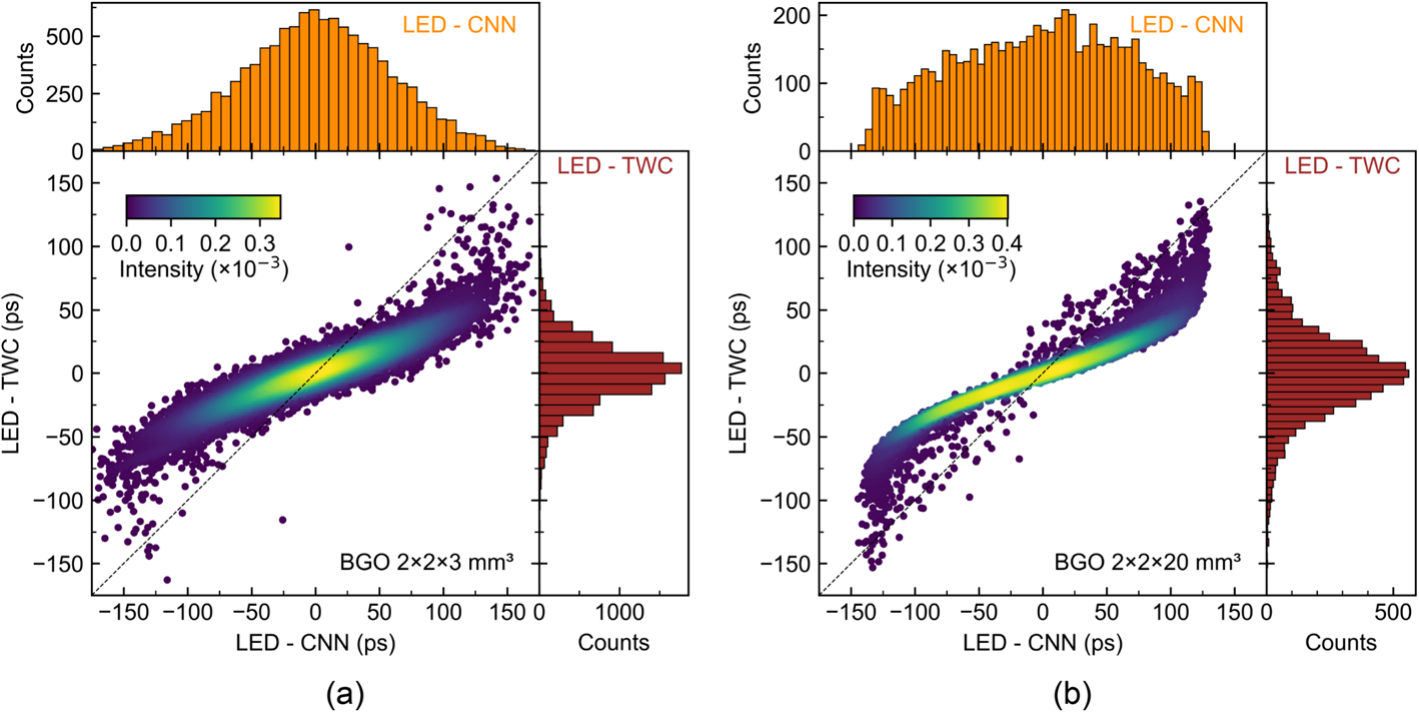
Scatter plots and marginal distributions of the difference in timing values between LED and CNN versus LED and TWC for (a) BGO ${2 \times 2 \times 3}\,\hbox {mm}^{3}$ and (b) BGO ${2 \times 2 \times 20}\,\hbox {mm}^{3}$

The difference between the timing values of the LED method to the two other methods that correct the LED is depicted as a scatter plot and marginal distributions in Fig. 4. A perfect diagonal trend in the scatter plot would signify no disparity in the correction applied by the TWC and CNN. For both BGO dimensions, LED-CNN values exhibit a wider spread compared to LED-TWC, suggesting that the CNN corrects more aggressively events found in the tails of the timing distributions. In the case of BGO ${2 \times 2 \times 20}\,\hbox {mm}^{3}$, the LED-CNN difference distribution is flatter than the LED-TWC counterpart, indicating a more uniform correction of all coincidence events.

The difference in the calculated $\hbox {CTR}_{\text {eq}}$, when labeling of the coincidence events is assumed versus when no labeling is assumed (Eq. (4) vs. (5)), is displayed in Table 2 for all timing methods. We remind that these equivalent CTR calculations are limited to the task of detecting a hot spot in a uniform cylinder [31]. Labeling the TOF events yields a CTR improvement of less than 10 % for BGO ${2 \times 2 \times 3}\,\hbox {mm}^{3}$, but in the range of 20 % for BGO ${2 \times 2 \times 20}\,\hbox {mm}^{3}$.

**Table 2:** Equivalent CTR with unlabeled vs labeled events

Scintillator	Method	$\hbox {CTR}_{\text {eq}}$		
		Unlabeled	Labeled	Gain
		(ps)	(ps)	(%)
BGO ${2 \times 2 \times 3}\,\hbox {mm}^{3}$	LED	184 ± 2	171 ± 2	7 ± 1
	TWC	155 ± 1	142 ± 2	8 ± 2
	CNN	138 ± 1	127 ± 1	8 ± 1
BGO ${2 \times 2 \times 20}\,\hbox {mm}^{3}$	LED	436 ± 6	357 ± 6	18 ± 2
	TWC	399 ± 6	319 ± 6	20 ± 2
	CNN	389 ± 7	313 ± 6	20 ± 2

Different CNN architectures and training parameters, distinct from those outlined in Sec. 2.2 that were employed to generate the results presented in this study, were also tested. These included the exploration of architectures with two to four convolutional layers, using different numbers of filters (ranging from 32 to 128) in these layers, employing larger filter sizes (up to 2x50 for the first convolutional layer and up to 1x30 for subsequent layer(s)), disabling max pooling, and introducing dropout with a 50 % probability before the last fully-connected layer. These tests revealed a CTR performance of the CNN within the error bars of the values presented in this study.

## Discussion

The resurgence of interest within the last decade for BGO in TOF-PET detectors is spurred by the enhanced detection of its Cherenkov photons, thanks to novel fast and low-noise SiPMs with improved blue-UV sensitivity. However, the slow scintillation light of BGO generates signals with larger time jitter adversely affecting CTR, but this shortcoming can be mitigated by refining the timing estimation. The optimization of timing estimators to leverage signal shapes or even labeling events based on their timing quality has shown potential benefits for timing estimation [14, 32] and consequent potential improvements of SNR in reconstructed images [31].

In this study, it was observed for BGO ${2 \times 2 \times 20}\,\hbox {mm}^{3}$ that a time-walk correction using two points on the rising edge produces a similar improvement in $\hbox {CTR}_{\text {FWHM}}$ as a deep learning approach requiring the complete digitization of the rising edge (Fig. 2 and 3, and Table 1). We even focused on optimizing the CNN’s performance by using a highly precise digitization (Sec. 2.2). Nonetheless, the CNN demonstrated superior performance in reducing tails of the coincidence time distribution and enhancing the $\hbox {CTR}_{\text {FWTM}}$ and $\hbox {CTR}_{\text {eq}}$. The current work had a primary emphasis on achieving the best possible outcome in evaluating timing performance limits with a fine digitization. Future work could explore alternative (coarser) digitization, aiming for a broader waveform amplitude range, and investigate the inclusion of other signal features (amplitude, rise time, etc.) that could further enhance the information fed into the CNN. This could help, along with acquiring a larger number of waveforms, to better explain the limited improvement observed for longer BGO crystals with the CNN and to evaluate whether improved CTR can be achieved by incorporating more waveforms and additional parts of each waveform into the training. Additionally, the TWC was done using a linear fit function (Eq. (2)) as in [15], but more sophisticated functions could also enhance TWC even further. This will be tested in a future work.

Given the smaller number of waveforms for BGO ${2 \times 2 \times 20}\,\hbox {mm}^{3}$ compared to BGO ${2 \times 2 \times 3}\,\hbox {mm}^{3}$ after event selection (Sec. 2.1), we tried augmenting the dataset with waveforms obtained from an additional measurement with the bench. This provided no significant improvement in CNN performance, indicating that the original dataset likely already contained sufficient data. We also conducted waveform acquisition for CTR measurements using ${2 \times 2 \times 3}\,\hbox {mm}^{3}$ and ${2 \times 2 \times 20}\,\hbox {mm}^{3}$ LSO:Ce:0.2%Ca crystals. For those detectors, it appears that the information is well contained in a single point by LED, as the CNN with the architecture described in Sec. 2.2 showed negligible $\hbox {CTR}_{\text {FWHM}}$ improvement, from 61 ps to 59 ps for 3-mm-long LSO and from 108 ps to 107 ps for 20-mm-long LSO. Other novel detectors—currently in the early stages of exploiting energy sharing or unconventional scintillation mechanisms [33–40]—could also be pertinent to test with deep learning since their signals could be rich in features.

Implementing a deep learning model for TOF estimation in a multi-channel system is more demanding than using TWC. Yet, there are efforts to enable direct real-time usage of machine learning in PET detectors, for instance with artificial neural networks (ANNs) implemented on field programmable gate arrays (FPGAs) to recover triple coincidences [41] or perform time discrimination [42]. Source position prediction was also recently evaluated with an ANN implemented directly on chip [43]. Endeavours were also made to implement CNNs on FPGAs [44] and recent FPGAs show great outlook for enhanced real-time analysis, for instance, in the treatment of PET data [45]. Overall, the following years might highlight more and more how it is important to include machine learning methods at the sensor’s level [46], and not only for offline analysis.

The question also remains whether the quality of information in signals can be preserved in multi-channel systems to fully benefit from deep learning model dedicated for TOF estimation. For instance, the detectability of timing features in signals is anticipated to diminish due to a general decrease in performance with scintillator arrays in contrast to the optimized single-channel setup employed in the current study. This may arise from factors such as light crosstalk [47] or front-end limitations of ASICs [48], among others. Recent impressive results achieved by machine learning models in diverse multi-channel detector geometries, including light-sharing arrays [49], semi-monolithic [50], and monolithic crystals [51], particularly in the context of interaction positioning, nonetheless suggest some promise for near-future developments.

In order to achieve optimal CTR, this precise positioning can also be necessary to mitigate any position-dependent time biases, notably from depth-of-interaction (DOI) [20, 28, 52, 53]. While challenging to assert with CNNs, it may be unnecessary to employ a dedicated DOI estimator to improve timing. Instead, employing a CNN-based TOF estimator, but also a TWC-based estimator, could intrinsically correct DOI timing biases contained in the signals. Nevertheless, it remains to be shown how to perform a timing estimation with a mixture of factors, such as DOI and Cherenkov photon fluctuations [20, 32]. A recent work also has shown the potential of a CNN-based TOF estimation in simulations of monolithic crystals where the spatio-temporal distribution of scintillation photons on a SiPM array was used for training [54].

The current study highlights the advantages of HF electronics in preserving crucial features within analog waveforms, presenting opportunities for exploitation by both the TWC and CNN methods. There are ongoing efforts in lowering the power consumption and scalability of the HF readout [16, 48, 55, 56]. In contrast to traditional analog readout methods, emerging fully digital photodetectors [57, 58] with the capability for numerical time-tagging of individual photons hold promise for substantial CTR improvements, particularly at good SPTR values. Using the first detected photon of a digital SiPM having a potential forthcoming 10 ps FWHM SPTR, Monte Carlo simulations predicted CTR values of $\sim$30 ps and $\sim$122 ps FWHM for 3 mm and 20 mm long BGO, respectively, whereas simulations with an analog counterpart yielded values above 100 ps and 200 ps FWHM for the two crystal lengths [7]. The application of deep learning in a digital framework capable of fully preserving the arrival time distribution of photons emerges as a potential avenue for pushing the limits of CTR. Non-deep learning methods could also make usage of this photon distribution to achieve excellent CTR [21].

Finally, more investigation is needed on deep learning models and training strategy applied for TOF estimation and CTR improvement. The method in this study used a combination of a CNN with the LED information for the TOF estimation. This has the great advantage of providing unbiased estimation compared to a training done with an extensive dataset of multiple source positions (especially at the edges of the acquisition space), and only needing a single source position acquisition [24]. This method was able to reduce the tails of the BGO timing spectrum, therefore reducing the effective variance that can be strongly affected by these tails. This reduction, which is expected to persist with new SiPMs better able to detect Cherenkov photons, therefore places BGO in good position against lutetium-based (e.g., LYSO, LGSO) scintillators which still have better light yield [59, 60] and CTR [7, 61], but suffer from higher cost and lower stopping power [17]. It remains to be seen if the conclusion of the present study—showing almost equivalent performance improvement with a CNN and a simpler TWC method—will remain since high-performance readouts could raise the possibility of detecting more information in the signals to further improve TOF resolution.

## Conclusions

The timing performance of detectors for TOF-PET imaging can be improved with a better exploitation of features imprinted by the interaction process in the signal rising shape. In this work, we have presented a comparison of the CTR improvements in BGO scintillators either by using two-threshold–based time walk correction or by using a convolutional neural network (CNN) trained on digitized rising edges of waveforms, in contrast to a traditional leading edge discrimination approach. A timing setup with recently developed NUV-HD-MT SiPMs read out by high-frequency electronics was used to assess the full potential and CTR limits of BGO. For ${2 \times 2 \times 3}\,\hbox {mm}^{3}$ crystals, the CTR can reach 129 ± 2 ps FWHM with time walk correction and 115 ± 2 ps FWHM with the CNN, representing a gain of 18 % and 26 % against a standard leading edge discrimination estimator, respectively. For longer ${2 \times 2 \times 20}\,\hbox {mm}^{3}$ crystals, the CTR of the two methods is similar (both around 240 ps FWHM) with a $\sim$15 % gain from the standard timing estimator, although with slightly better tail suppression in the coincidence time histogram when using the CNN. The higher implementation complexity for a full waveform digitization used for the CNN could therefore be alleviated with the simpler two-threshold approach that seems to provide most of the information necessary to improve the CTR for longer BGO crystals. Future studies could focus on novel network architectures and necessary digitization granularity for efficient feature detection to find the optimal trade-off between CTR, complexity and power consumption, especially for complete multi-channel TOF-PET systems.

## Data Availability

The datasets used and/or analyzed during the current study are available from the corresponding author on reasonable request.
